# Space Radiation Risks for Astronauts on Multiple International Space Station Missions

**DOI:** 10.1371/journal.pone.0096099

**Published:** 2014-04-23

**Authors:** Francis A. Cucinotta

**Affiliations:** Department of Health Physics and Diagnostic Sciences, University of Nevada Las Vegas, Las Vegas, Nevada, United States of America; Belgian Nuclear Research Centre SCK/CEN, Belgium

## Abstract

Mortality and morbidity risks from space radiation exposure are an important concern for astronauts participating in International Space Station (ISS) missions. NASA’s radiation limits set a 3% cancer fatality probability as the upper bound of acceptable risk and considers uncertainties in risk predictions using the upper 95% confidence level (CL) of the assessment. In addition to risk limitation, an important question arises as to the likelihood of a causal association between a crew-members’ radiation exposure in the past and a diagnosis of cancer. For the first time, we report on predictions of age and sex specific cancer risks, expected years of life-loss for specific diseases, and probability of causation (PC) at different post-mission times for participants in 1-year or multiple ISS missions. Risk projections with uncertainty estimates are within NASA acceptable radiation standards for mission lengths of 1-year or less for likely crew demographics. However, for solar minimum conditions upper 95% CL exceed 3% risk of exposure induced death (REID) by 18 months or 24 months for females and males, respectively. Median PC and upper 95%-confidence intervals are found to exceed 50% for several cancers for participation in two or more ISS missions of 18 months or longer total duration near solar minimum, or for longer ISS missions at other phases of the solar cycle. However, current risk models only consider estimates of quantitative differences between high and low linear energy transfer (LET) radiation. We also make predictions of risk and uncertainties that would result from an increase in tumor lethality for highly ionizing radiation reported in animal studies, and the additional risks from circulatory diseases. These additional concerns could further reduce the maximum duration of ISS missions within acceptable risk levels, and will require new knowledge to properly evaluate.

## Introduction

In this paper we discuss predictions of risks and the probability of causation (PC) also known as attributable risk for space radiation exposure for astronauts participating in one or more missions on the International Space Station (ISS). In low Earth orbit (LEO), astronauts are exposed to galactic cosmic rays (GCR) – made up of high-energy protons and high-energy and charge (HZE) nuclei, protons and electrons in Earth’s radiation belts, and solar particle events (SPEs) – comprised largely of low- to medium-energy protons. The lack of human epidemiology data for these radiation types makes risk predictions highly uncertain. Also, the risks of low dose radiation for circulatory disease and early and late effects in the central nervous system are an emerging concern for space travel. NASA limits astronaut exposures to a 3% risk of exposure induced death (REID) for cancer, and protects against uncertainties in risks projections using an assessment of 95% confidence intervals (CI) of risk estimates [1]–[3]. NASA has followed recommendations from the National Council of Radiation Protection and Measurements (NCRP) for setting astronaut radiation limits [4], [5]. In addition, improvements in other areas of spaceflight safety and ground-based occupational safety [6]–[8] should be considered in the context of radiation risk limits. The principle of As Low As Reasonably Achievable (ALARA) is a requirement to limit exposures even below radiation limits [4], [5].

The uncertainties in estimating risks and dose limits for astronauts have been recognized by several reports from the National Research Council (NRC) [9]–[11] and NCRP [5], [12]. The uncertainty is largely due to the lack of information on the radiobiology of GCR HZE particles that produce distinct biological effects from ground-based radiation such as X-rays or gamma rays, and for which no human data is available. This uncertainty led the NCRP to recommend that methods used for LEO radiation risk assessments [5] are not of sufficient accuracy for long-term exposure to GCR, and to a full NCRP report [12] advising NASA on the information needed to accurately estimate risks and set exposure limits. In the face of the obstacles of large radiobiological uncertainties while needing to support current missions and future mission planning, NASA developed an approach to estimate the 95% CL in cancer risk estimates using an approach developed by the NCRP [12], [13] that was extended to the space radiation exposures [2], [14], [15]. This approach formed the basis for the current NASA radiation exposure limits [1] and supported by a NRC review [10].

Experimental studies have shown that HZE nuclei produce both qualitative and quantitative differences in biological effects compared to terrestrial radiation [5], [12], [16]–[23], leading to large uncertainties in predicting exposure health outcomes to humans. Animal studies of tumor induction by HZE particles have shown high values for relative biological effectiveness (RBE) and qualitatively distinct dose responses [16]–[19] compared to gamma rays, higher rates of metastatic tumors [19], [20] and increases in tumor grade [21], [22]. The shape of the dose response for tumor induction by HZE particles is similar to older studies with fission neutrons which suggested more lethal tumors were induced compared to gamma rays [23], [24]. Concepts from the multi-stage model of cancer development suggest that HZE particles likely affect both tumor initiation and promotion with higher frequency compared to gamma rays, and that non-targeted effects [25]–[27] play an important role at the doses to be incurred on space missions. Qualitative differences in cancer risks are not accounted for in current uncertainty assessment by NASA [2], [11]. In this report I describe sensitivity analysis on how increased tumor lethality for highly ionizing particles could modify risk estimates for ISS missions. The potential impacts of qualitative differences between HZE particles and low LET radiation were not considered in our earlier reports [2], [3].

Beyond efforts related to risk limitation prior to a mission, there is a concern for cancers observed in crew member’s post-mission and their possible association with prior space radiation exposures. The PC is a conditional probability of risk used as an indicator of a potential causal relationship between radiation exposure and occurrence of cancer in a population. PC estimates above 50% suggest an observed cancer was more likely to be attributed to radiation exposure than not [28]–[30]. The calculation of PC with concomitant uncertainty analysis provide an estimate that an observed cancer was caused by occupational exposure, however, should be augmented with considerations of an individual’s family history of disease, possible individual exposure to other carcinogens, and of individual based biomarkers.

In the remainder of this report, I first summarize the NASA cancer risk assessment model (denoted as NSCR-2012) [2] and its application to PC estimates and years of expected life-loss for ISS missions. Predictions focus on an individual that would participate in multiple ISS missions where radiation limits may be approached or projected to be exceeded, including current interest in 1-year missions on the ISS. For a reasonable worse-case, a 1-year mission is considered near the average solar minimum combined with other 6-month missions to the ISS in other times of a solar cycle. The next solar minimum should occur around 2019 to 2021, however the depth of the solar modulation is difficult to predict at this time with the most recent solar minimum in 2009–2010 one of the deepest on record [2]. Previously [3] results of a meta-analysis of circulatory diseases in populations exposed to low LET radiation [31] and methods to extrapolate these results for space radiation exposures [3], were used in REID estimates of ischemic heart disease (IHD) and cardiovascular disease (CVD), and to make predictions of the combined REID for cancer and circulatory diseases for space missions. In this report, I introduce a method to revise the uncertainty analysis in the NSCR-2012 model [2], [3] to study the effects of increased tumor lethality for highly ionizing radiation. For the first time, risk predictions for astronauts participating in 1-year or multiple ISS missions are made, including sex and age specific comparisons for an average U.S. population and a population of never-smokers (NS) [2], [32].

## Methods

### Cancer Risk Projection Model

The instantaneous cancer incidence or mortality rates, λ_I_ and λ_M_, respectively are modeled as functions of the tissue averaged absorbed dose *D_T_*, or dose-rate *D_Tr_*, sex, age at exposure *a_E_*, and attained age *a* or latency *L,* which is the time after exposure *L = a-a_E_*. The λ_I_ (or λ_M_) is a sum over rates for each tissue that contributes to risk, λ_IT_ (λ_MT_). These dependencies vary for each cancer type that could be increased by radiation exposure. The total risk of exposure induced cancer (REIC) is calculated by folding the instantaneous radiation cancer incidence-rate with the probability of surviving to time *t,* which is given by the survival function *S_0_(t)* for the background population times the probability for radiation cancer death at previous time, summing over each mission exposure, and then integrating over the remainder of a lifetime:
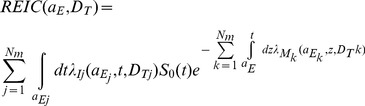
(1)where z is the dummy integration variable. In Eq. (1), N_m_ is the number of missions (exposures), and for each exposure, *j*, there is a minimum latency of 5-years for solid cancers and circulatory diseases, and 2-years for leukemia assumed. After adjustment for low dose and dose-rates through introduction of the dose and dose-rate effectiveness factor (DDREF) and radiation quality through the NASA defined quality factor (QF) function, the tissue-specific cancer incidence rate for an organ dose equivalent, *H_T_*, can be written as a weighted average of the multiplicative and additive transfer models, often called a mixture model:
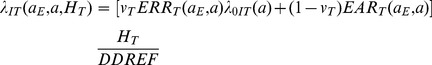
(2)where ν*_T_* is the tissue-specific transfer model weight, λ_*0I_T_*_ is the tissue-specific cancer incidence rate in the reference population, and where *ERR_T_* and *EAR_T_* are the tissue specific excess relative risk and excess additive risk per Sievert (Sv), respectively, that are described in other reports [33]–[35].

### Space Radiation Organ Dose Equivalent

For calculations of space radiation tissue specific cancer risks, Eq. (2) is used for the cancer incidence risk rate with the organ dose equivalent estimated using the NSCR-2012 model [2], [3]. The NASA QF depends on two physical parameters: particle charge number, Z and kinetic energy per nucleon, E. However, a key parameter that describes the density of a particle track is Z^*2^/β^2^ where Z* is the effective particle charge number and β is the particle velocity scaled to the speed of light, is used to simplify the description [2]. In the NASA approach [2] distinct QFs for estimating solid cancer and leukemia risk are used, Q_solid_ and Q_leukemia_, respectively. Here a cancer risk cross section representing the biological effect probability per particle is written as [2]:
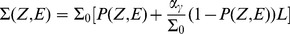
(3)


With *L* the particle LET, and
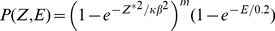
(4)where the central estimates of the three parameters of the model (Σ_0_/α_γ_, *m*, and *κ*) that are listed in Table 1 are estimated based on subjective estimates of results from radiobiology experiments [2]. A QF function is then defined by

**Table 1:** Parameters for central estimates of NASA quality factor (QF) parameters from Eq.’s (3) to (5) for solid cancer and leukemia risks.*

*Parameter*	*Solid Cancer*	*Leukemia*
***m***	3	3
***κ***	550 (1000)	550 (1000)
***Σ_o_/α_γ_µm^2^ Gy***	7000/6.24	1750/6.24
***E_TD._ MeV/u***	0.2	0.2

*Values in parenthesis for when distinct values for light ions (Z ≤ 4) are to be used.



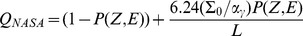
(5)For calculations for a specific GCR particle type described by *Z* and *E*, Eq. (2) is replaced by
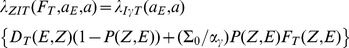
(6)where λ_γI_ is the inner bracketed terms in Eq. (2) that contains the ERR and EAR functions for individual tissues. Calculations are made using the NASA models of the GCR environments and radiation transport in spacecraft materials and tissue, which estimate the energy spectra, *φ_k_(E)* of each particle *k* for 190 isotopes of the elements from Z = 1 to 28, neutrons, and contributions from pions, electrons, muons, and gamma rays [2]. The model agrees with flight measurements for dose and dose equivalent to within ±15% as shown in prior reports [2], [36]–[38], which is accounted for in the model uncertainty analysis. The fluence spectra, *F(X_tr_)* where *X_tr_* = Z^*2^/β^2^ can be found by transforming the energy spectra, φ*_k_(E)* for each particle, *k* of mass number and charge number, *A_k_* and *Z_k_* respectively as:



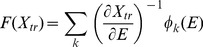
(7)The Jacobian in Eq. (8) is evaluated using the Barkas [39] form for the effective charge number given by
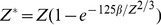
(8)


The tissue specific cancer incidence rate for GCR or SPEs can then be approximated by:
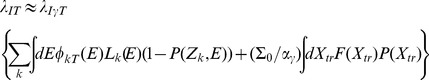
(9)where *L_k_(E)* is the LET of particle *k*, and the summation is over all particles types contributing to exposure. A 50% percentile M or F anatomical model is used in organ dose evaluations [36]. A summation over all cancer types is made in evaluating the radiation contribution to the survivor function in evaluating tissue specific risks, and a further summation over all cancer types to evaluate the over-all cancer risk.

### Hazard Rate for Cancer Mortality

The Hazard rates for cancer mortality *λ_M_* are modeled following the method introduced by the U.S. National Academy of Sciences, Committee on the Biological Effects of Ionizing Radiation (BEIR VII) report [34], whereby the incidence rate of Eq.(2) is scaled by the age, sex, and tissue specific ratio of rates for mortality to incidence in the population under study:
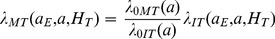
(10)


### Qualitative Differences Due to Increased HZE Particle Tumor Lethality

Studies of tumors in mouse and rats [16]–[24] by highly ionizing radiation, suggest that solid tumors induced by HZE particles are qualitatively distinct from background tumors or tumors induced by low LET with observations of higher tumor grade, and increased propensity for metastatic tumors. An upper limit on the possibility of higher tumor lethality would be to use REIC estimates for REID estimates on space missions. However this estimate would be too large due to the presence of low LET particles such as protons that make up a significant fraction of space radiation organ doses. To make a more realistic estimate we assume the increased lethality follows a radiation quality dependence similar to the RBE for cancer induction. The cancer mortality rate is modified as.
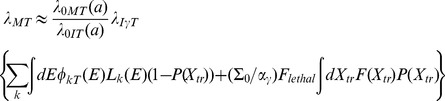
(11)where the second term in Eq. (11) is increased by a tumor lethality fraction, *F_lethal_* for highly ionizing particles. The first term in Eq. (11) dominates for low LET radiation and is not altered under our considerations of increased tumor lethality for highly ionizing radiation. For our sensitivity study of *F_lethal_* we considered a probability distribution function (PDF) to represent the uncertainty in the increased lethality for HZE particles and secondary particles from neutrons. The PDF is modeled as a normal distribution considering median values of 1.25 or 1.5 and using a 25% variance, and compared to results without any increase in tumor quality for densely ionizing radiation.

### Expected Years of Life-Loss and Probability of Causation

The tissue specific life-loss expectancy, *LLE_T_* for cancer death from radiation exposure can be estimated in terms of conditional survival probabilities for the unexposed and exposed populations:
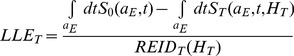
(12)


The PC is the fraction of the incidence of a disease in a population (exposed and non-exposed) that is due to radiation exposure. Thus the PC represents the incidence of a disease in the population that would be eliminated if there were no radiation exposure. The PC is estimated from Eq. (1) by limiting the upper limit of integration to the year of disease diagnosis, *a_Diag_* for both the exposed population and the reference population, with the PC defined in terms of the conditional tissue specific excess relative risk, *ERR_T_*:
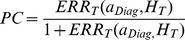
(13)where the conditional *ERR_T_* is defined by
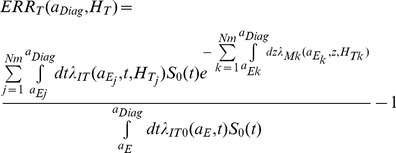
(14)


### Uncertainties for Multiple ISS Missions

The NSCR model considers PDFs to represent the uncertainties in values for the QF, DDREF, epidemiology data (statistical, bias, and dosimetry errors) and transfer weights for assigning proportions assigned to additive and multiplicative models, and the physics uncertainties in organ dose evaluation. PDFs describing these uncertainties were described in our earlier reports [2], [3]. Monte-Carlo sampling over the combined model is implemented using standard methods [2], [13].

For evaluation of multiple ISS missions, for each Monte-Carlo trial the random deviates representing the QF, DDREF and epidemiology and transfer uncertainties are held fixed over all missions considered for a given astronaut career. However, in considering physics uncertainties related to particle spectra and doses at specific organs, assuming these uncertainties are correlated across all missions or vary from mission to mission could be considered. Factors that arise include systematic errors in organ dose evaluation or use of retrospective dosimetry, or possible errors in future mission projections with an uncertainty that varies for each prediction. Because the physical uncertainties are much smaller than the QF and DDREF uncertainties, these choices lead to nearly identical results. We assumed the physical uncertainties were independent for each mission in the predictions described next.

## Results

**Figure 1:**
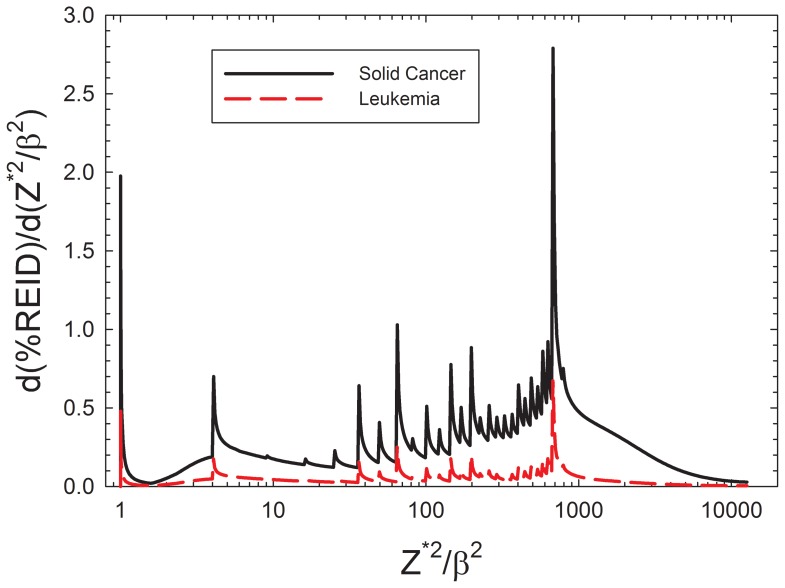
Distribution of the percent risk of exposure induced death (%REID) for solid cancer and leukemia versus particles type as represented by Z*^2^/β^2^ for 45-y female never smokers. Results are for exposure to space radiation on an 1-year ISS mission near solar minimum with 20 g/cm^2^ aluminum shielding.

We considered an array of risk calculations for female and male astronauts with ages between 40 and 50 years at the time of an ISS mission. Calculations are made for average of either solar minimum, solar maximum or median solar cycle conditions for a 51.6×380 km circular orbit. Solar modulation parameters for these environments were described in our earlier report [2]. Calculations assume an average shielding of the ISS is equivalent to 20 g/cm^2^ of aluminum based on previous analysis [34]. Trapped radiation exposures decrease rapidly with increasing depth and GCR dominated organ exposure at the average or larger shielding depths, and change modestly with increasing shielding beyond 20 g/cm^2^ [2], [3]. The distribution spectra of %REID per year for solid cancer and leukemia versus the radiation quality descriptive parameter, Z^*2^/β^2^, is shown in **Figure 1** for average spacecraft shielding conditions for a 40-y female NS with average solar minimum conditions. The parameter Z^*2^/β^2^ describes the relationship between biological effectiveness and particle track more effectively than LET and is used differentially in the NASA quality factor for light (Z≤2) and heavy particles (Z>2) [2]. A prominent peak occurs near 26^2^ corresponding to relativistic iron particles with similar peaks near values of integer Z^2^ observed for other HZE particles. However, the REID distribution at large values of Z^*2^/β^2^ (>100) also includes contributions from stopping hydrogen and helium particles produced as secondary radiation inside spacecraft or tissue through nuclear reactions and atomic slowing down. **Figure 1** illustrates that HZE particles and high LET secondaries produced by neutrons and the slowing down of charged particles dominate cancer risks for ISS missions.

**Figure 2:**
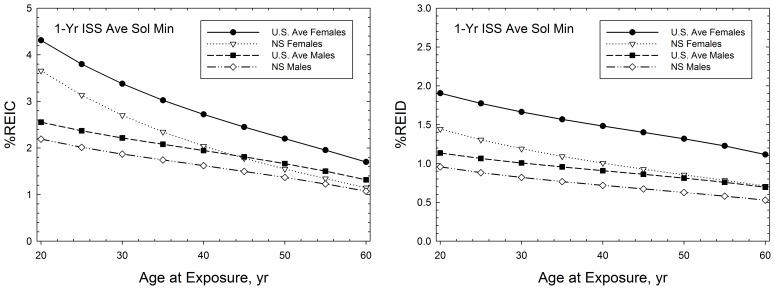
Central estimates of the percent risk of exposure induced cancer (% REIC) (left panel) and percent risk of exposure induced death (%REID) (right panel) versus age at exposure for 1-year ISS missions near solar minimum. Predictions for F and M of the U.S average population or a never-smoker (NS) population are shown.

In order to illustrate the age dependence of REIC and REID predicted by the NSCR-2012 model for F and M populations, **Figure 2** shows central estimates of REID from cancer versus ages of exposure from 20 to 60 y for 1-year ISS missions for average solar minimum conditions. **Figure 2** includes results for average U.S. and NS population. Our previous report described our NS model to represent most astronauts that are life-time abstinent from the use of tobacco products [2]. The higher risks for the U.S. population compared to the NS populations is an important considerations for crew selection when considering possible maximum mission duration within NASA safety standards.

**Table 2** shows organ average dose equivalents for solid cancer, leukemia and circulatory disease risk estimates. Results are shown for 1-year exposure for three solar conditions corresponding to the average solar maximum, solar minimum, and solar median for a fixed altitude of 380 km. In practice the ISS would possibly orbit the Earth at a slightly higher altitude near solar maximum and lower altitude near solar minimum. GCR dominate organ dose equivalent over trapped radiation exposure for altitudes of at least 400 km and vary by only a few percent for nearly circular orbits within a few 10′s of km altitude of 380 km. At higher altitudes (>400 km) the trapped proton contributions to organ dose equivalents become appreciably larger and would increase total exposures by a significant amount.

**Table 2:** Summary of International Space Station (ISS) organ dose equivalents for solid cancer, leukemia and circulatory disease risk estimates for different solar cycle conditions for females (males).

Missions	Solid Cancer, Sv	Leukemia, Sv	Circulatory Disease, Gy-Eq
**1-Y Solar Min**	0.187 (0.175)	0.109 (0.104)	0.132 (0.126)
**1-Y Solar Med**	0.146 (0.138)	0.084 (0.08)	0.10 (0.096)
**1-Y Solar Max**	0.10 (0.094)	0.054 (0.052)	0.072 (0.064)
**1-Y Solar Min and 0.5-Y Solar Med**	0.26 (0.244)	0.151 (0.144)	0.182 (0.174)
**1-Y Solar Min, 0.5-Y Solar Med, and 0.5-Y Solar Max**	0.31 (0.291)	0.178 (0.171)	0.215 (0.205)

Predictions are for single or multiple ISS missions. Solar cycle conditions considered are average solar minimum (Solar Min), average solar maximum (Solar Max), or median solar cycle (Solar Med), with solar modulation parameters for these conditions described in [2].

**Figure 3:**
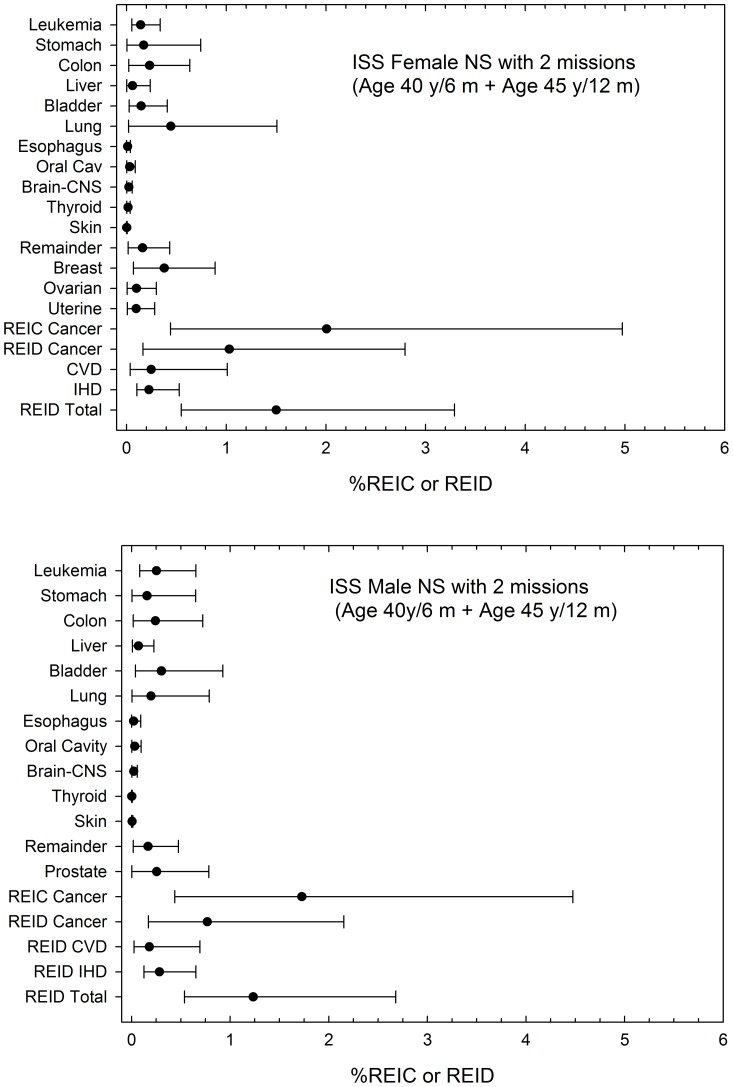
Predictions of tissue (disease) specific percent risk of exposure induced cancer (%REIC) or percent risk of exposure induced death (%REID) for participation in two ISS missions at age 40-y and age 45-y (6-months at solar median and 1-year near solar minimum) for F and M never-smoker populations.

Predictions of tissue specific REID, total cancer REID and the additional REID contributions from CVD and IHD are shown in **Figure 3** for the case of two ISS missions of total duration of 18 months (6 months for solar median conditions, and 12 months near solar minimum). Central estimates and upper 95% CI of REIC and REID for cancer are slightly reduced (∼2%) when CVD and IHD are included in calculations due to the role of competing risks. Risks for females are about 20% higher than males due to the added contributions from breast, ovarian, and uterine cancers, and an important difference in lung cancer risks for males and females. Also, organ dose equivalents for females are a few percent larger than males due to less body mass. Predictions of CVD and IHD are similar for M and F and NS and U.S. average populations [25], and increase overall REID by about 40% on average.

**Figure 4:**
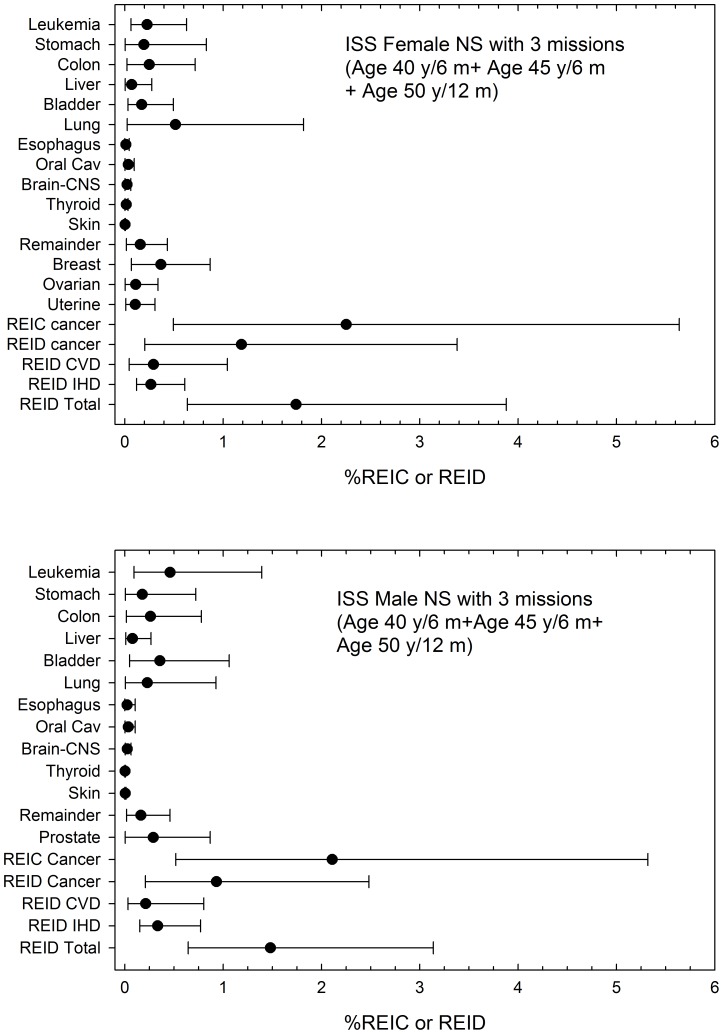
Predictions of tissue (disease) specific percent risk of exposure induced cancer (%REIC) or percent risk of exposure induced death (%REID) for participation in three ISS missions at age 40-, 45, and 50-y (6-month at solar median, 6-months near solar maximum, and 1-year near solar minimum) for F and M never-smoker populations.

**Figure 4** shows a similar comparison as **Figure 3** for astronauts participating in three ISS missions of total duration of 2-year with a 1-year mission near solar minimum at age 50-y. In this comparison upper 95% CIs exceed 3% for females and approach 3% for males for cancer alone. The inclusion of circulatory disease risk leads to a predictions where both males and females have upper 95% CIs above 3% with values close to 4% for females.

**Figure 5:**
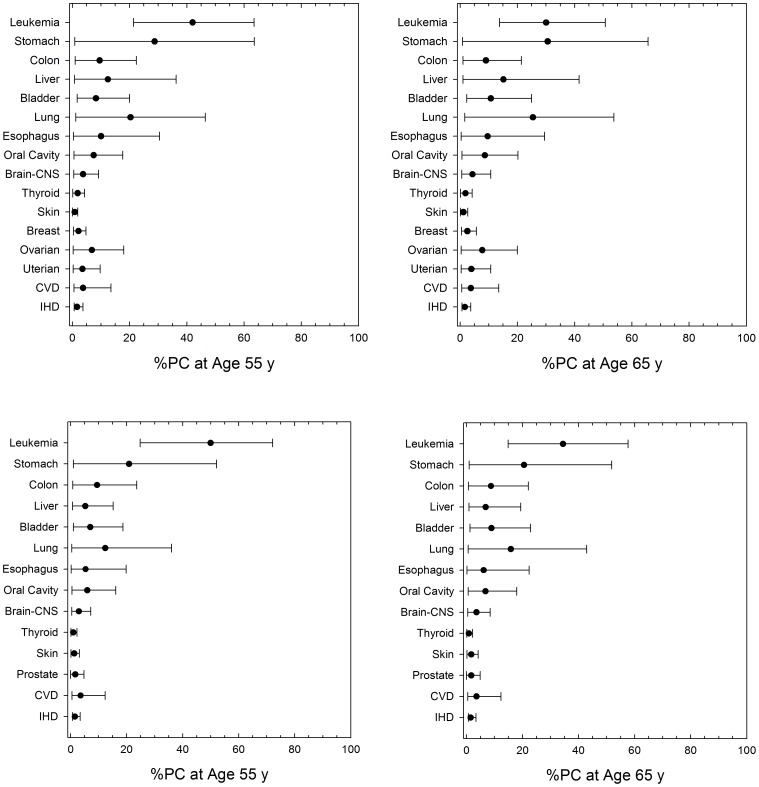
Predictions of tissue (disease) specific %PC at age 55-y (left panel) and age 65-y (right panel) following participation in two ISS missions at age 40-y and age 45-y (6-month at solar median, and 1-year near solar minimum) for F and M never-smoker populations.

**Figure 6:**
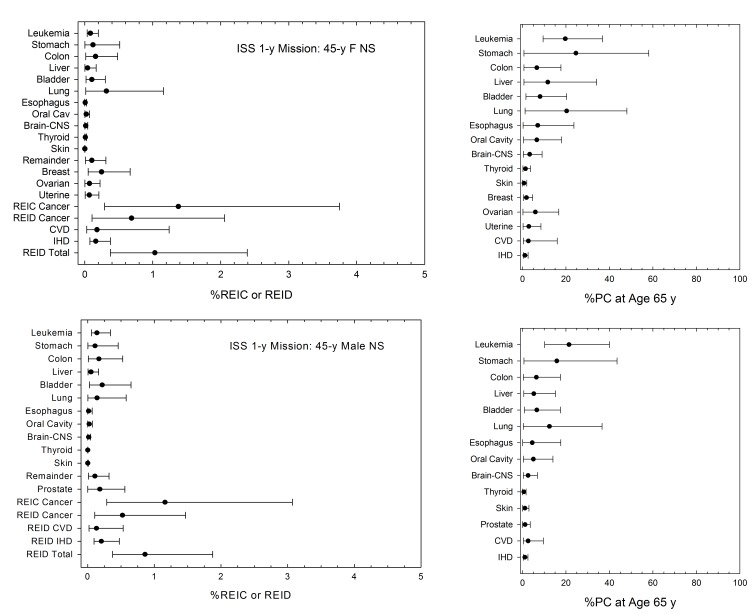
Predictions tissue (disease) specific percent risk of exposure induced cancer (%REIC) or percent risk of exposure induced death (%REID) or percent probability of causation (%PC) at age 65-y (right panels) following participation in1-y ISS missions at age 45-y for F and M never-smoker populations.

**Figure 5** shows predictions of the PC following participation in two ISS missions corresponding to the results of **Figure 3**. Results are shown for F and M NS with disease diagnosis of age 55-y and 65-y which correspond to 15- and 25-y after the first ISS mission, and 5- and 15-y after a 1-year ISS mission, respectively. By age 65-y, %PC at the upper 95% confidence interval exceed 20% for leukemia, stomach, colon, lung, esophageal and ovarian cancers. %PC for circulatory diseases are modest (<10%) because of the large background and longer latency that results from epidemiology analysis compared to radiation induced cancers. %PC for leukemia’s decline at older ages while %PC for solid cancers, CVD and IVD increase modestly at older ages (>65 y) (result not shown). Similar results for 1-year missions with no other space exposures for mission age of 45-y and PC estimates at age 65-y are shown in **Figure 6**. These results suggest a 1-y mission near solar minimum would not exceed NASA radiation limits for astronauts with no prior occupational radiation exposures. Our predictions at solar minimum support that 1-year missions at any time in a solar cycle are below limits. However, other prior occupational exposures and changes to risk assessment models may alter this conclusion. **Table 3** and **4** also show the average years of life-loss expectancy (LLE) if death from space radiation exposure would occur. Leukemia has a LLE of about 23-y, overall cancer 15-y, and specific solid cancers varying from 12- to 20-y of life-loss. The LLE for circulatory diseases is less than that of cancer with values between 9- and 10-y resulting from our analysis.

**Table 3:** Predictions for female never-smokers participating in multiple ISS missions of organ dose equivalents (H_T_) and average loss of life expectancy (LLE) if radiation induced death occurs.

	Two ISS Missions for total duration of 18 m		Three ISS Missions for total duration of 24 m	
Tissue	H_T_, Sv or Gy-Eq	LLE, y	H_T_, Sv or Gy-Eq	LLE, y
**Leukemia, Sv**	0.151	23.1	0.179	24.9
**Stomach**	0.235	16.3	0.281	15.5
**Colon**	0.261	16.7	0.311	16.1
**Liver**	0.229	13.5	0.274	13.1
**Bladder**	0.231	11.2	0.276	11
**Lung**	0.264	13.2	0.314	12.8
**Esophagus**	0.249	15.1	0.297	14.6
**Oral Cavity**	0.308	15.3	0.365	14.6
**Brain-CNS**	0.286	18	0.339	17.2
**Thyroid**	0.308	22	0.365	20.8
**Skin**	0.282	11.8	0.336	11.7
**Remainder**	0.264	12	0.315	11.6
**Breast**	0.289	15.7	0.343	15.2
**Ovarian**	0.241	17.9	0.287	17.1
**Uterine**	0.241	17.1	0.287	16.4
**Total Cancer**	0.244	15	0.29	14.8
**CVD, Gy-Eq**	0.182	9.1	0.215	9.1
**IHD**	0.182	9.5	0.215	9.5

Results shown are for two missions at age 40, and 45 y of 180 d (at solar median), and 1-y (average solar minimum), respectively, or three missions at age 40, 45, and 50 y of 180 d (average solar maximum), 180 d (solar median), and 1-y (average solar minimum), respectively.

**Table 4:** Predictions for male never-smokers participating in multiple ISS missions of organ dose equivalents (H_T_) and average loss of life expectancy (LLE) if radiation induced death occurs.

	Two ISS Missions for total duration of 18 m		Three ISS Missions for total duration of 24 m	
Tissue	H_T_, Sv or Gy-Eq	LLE, y	H_T_, Sv or Gy-Eq	LLE, y
**Leukemia, Sv**	0.145	22.1	0.171	24.6
**Stomach**	0.227	15.6	0.272	14.9
**Colon**	0.251	16.4	0.299	15.7
**Liver**	0.235	14	0.281	13.5
**Bladder**	0.224	10.9	0.269	10.7
**Lung**	0.245	13.6	0.292	13.2
**Esophagus**	0.242	14.9	0.289	14.4
**Oral Cavity**	0.261	15.8	0.311	15.1
**Brain-CNS**	0.279	17	0.332	16.2
**Thyroid**	0.261	20.8	0.311	19.7
**Skin**	0.308	12	0.365	11.8
**Remainder**	0.253	11.7	0.302	11.3
**Prostate**	0.260	11.5	0.309	11.4
**Total Cancer**	0.228	15	0.272	15.6
**CVD, Gy-Eq**	0.174	9.8	0.205	9.8
**IHD**	0.174	10.6	0.205	10.6

Results shown are for two missions at age 40, and 45 y of 180 d (at solar median), and 1-y (average solar minimum), respectively, or three missions at age 40, 45, and 50 y of 180 d (average solar maximum), 180 d (solar median), and 1-y (average solar minimum), respectively.

**Figure 7:**
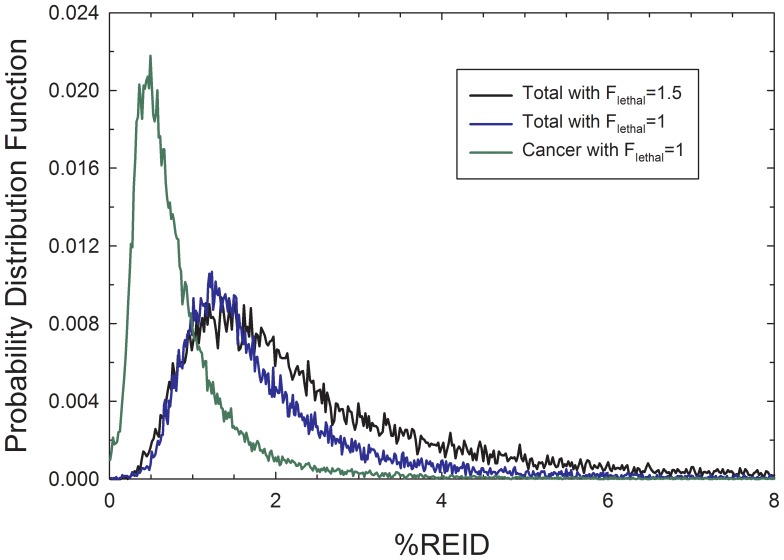
Predictions of the PDF for percent risk of exposure induced death (%REID) for cancer in NSCR-2012 model [2], and current results for the total (cancer and circulatory disease combined) and the modification of total %REID for the case of increased tumor lethality for highly densely ionizing particle. Predictions are for participation in two ISS missions at age 40-y and age 45-y (6-month at solar median and 1-year near solar minimum) for F never-smoker population.

**Figure 7** and **Table 5** show comparisons from results of sensitivity analysis of the role of increased lethality from HZE particles and nuclear secondaries using the adjusted mortality rate of Eq. (11). Results for 1-year ISS missions and two missions consisting of a 1-year mission at age 45 and a previous mission at age 40-y with median solar cycle conditions of 6-months are shown. The PDF for cancer in the NSCR-2012 model, total REID for cancer and circulatory disease risks combined, and total REID with inclusion of the enhanced tumor lethality assumption for densely ionizing particles with *F_lethal_* = 1.5 are shown in **Figure 7**. These results suggest that an increase in tumor lethality of 25 to 50%, which are values suggested by animal studies with high LET radiation, would reduce the mission duration for ISS missions with 95% CIs to be below the acceptable risk limits to less than 18 months. The combination of increased tumor lethality and addition of fatal circulatory disease risks could possibly impact crew participation on a 1-year mission within NASA’s radiation safety standards.

**Table 5:** Predictions of the increased percent risk of exposure induced death (%REID) for ISS missions due to potential qualitative difference leading to increases in tumor lethality for highly ionizing particles compared to tumors occurring in non-radiated populations or induced by low LET radiation. Results for F and M never-smokers are shown for cancer %REID and total %REID (cancer and circulatory diseases combined) assuming 20 g/cm^2^ average aluminum shielding.

Uncertainty Model	%REID (F) Cancer	%REID (F) Total	%REID (M) Cancer	%REID (M)Total
**1-y ISS mission at age 45 y**				
**NSCR-2012**	0.69 [0.11, 2.07]	1.03 [0.38, 2.37]	0.52 [0.11, 1.49]	0.86 [0.38, 1.9]
**F_lethal_ = 1.25**	0.78 [0.13, 2.89]	1.12 [0.4, 3.12]	0.59 [0.12, 2.09]	0.93 [0.40, 2.45]
**F_lethal_ = 1.5**	0.87 [0.16, 3.9]	1.21 [0.43, 4.0]	0.65 [0.14, 2.84]	0.99 [0.43, 3.08]
**6-m ISS mission at Age 40 y and 1-y ISS mission at age 45 y**				
**NSCR-2012**	1.01 [0.16, 2.79]	1.52 [0.55, 3.29]	0.77 [0.17, 2.15]	1.26 [0.54, 2.69]
**F_lethal_ = 1.25**	1.12 [0.2, 4.13]	1.59 [0.58, 4.41]	0.87 [0.2, 3.03]	1.32 [0.58, 3.48]
**F_lethal_ = 1.5**	1.25 [0.22, 5.46]	1.71 [0.61, 5.61]	0.96 [0.23, 4.02]	1.41 [0.62, 4.35]

Increases in tumor lethality is considered with the parameter, *F_letha_*_l_ as described by Eq.(11). The NASA Space Cancer Risk model (NSCR-2012) assumes *F_lethal_* = 1 with no uncertainty.

## Discussion

### Uncertainties in Risk Estimates

The detailed uncertainty analysis of radiation risks in this report lend support to the acceptable radiation risks for astronauts selected for 6-month or 1-year ISS missions. However because astronauts may have flown on prior ISS missions more detailed analysis was made for participation on multiple ISS missions. Any time spent on extra-vehicular activities (EVA) would marginally increase these results. However near solar maximum, the uncertainty in SPE occurrence is an important consideration, especially for EVAs if ISS orbits are within weak geomagnetic field locations or where trapped electron belt exposure occur, which could be increased by geomagnetic storms associated with SPEs. Other occupational exposures for astronauts, include possible participation in past space shuttle missions, medical diagnostic exposure related to fitness of duty and aviation exposures [40], [41]. The cumulative dose from these exposure increases with astronaut age. These exposures are not considered herein, but could raise estimates for individual crew members compared to the predictions described.

A major finding of our analysis is that female astronauts may exceed NASA radiation standards onward of 18 months in LEO and male astronauts onward of 24 months in LEO. Some variability in these times is expected due to solar cycle effects, other occupational radiation exposures, and the possibility of large solar particle events. Uncertainty reduction remains the most important area to improve risk predictions, extend allowable mission lengths, and ensure the safety of astronauts returning from long-term space missions. Improvements in the understanding of radiation quality and dose-rate effects could significantly reduce upper confidence intervals in the NSCR-2012 model. Improved information on specifying tissue specific transfer weights used in applying epidemiology data, and understanding differences in disease rates between model populations could also reduce uncertainties in risk estimates.

Human studies on circulatory disease risks are a more recent concern from epidemiology analysis of low dose radiation exposures [31], [42], [43]. For circulatory disease predictions the deterministic effects RBE model recommended by the International Commission on Radiological Protection (ICRP) is used as the central estimate [44], which as shown in Tables 2 and 3 leads to a lower GCR organ averaged equivalent dose compared to solid cancer risk and higher value compared to leukemia risk [3]. Uncertainty analysis for circulatory disease risk estimates were described in a recent report [3]. Concerns about a possible dose threshold for circulatory disease risks are an important consideration for ISS missions. Meta-analysis results that were based in-part to chronic exposures of radiation workers [31] suggests no dose threshold occurs. However, basic radiobiological understanding of how radiation increases circulatory disease risk at low dose and for highly ionizing radiation is lacking at this time. Life-style factors related to circulatory disease risk are expected to be larger compared to cancer risks, and also need to be considered in the understanding of possible radiation risks.

In the NSCR model a DDREF of 1.5 is used for solid cancers based on the BEIR VII report [34], and the recommendations of the NRC to NASA [11]. For circulatory disease risk predictions a DDREF is not applied because models were based on meta-analysis of several chronically exposed populations as described by Little et al. [31]. For cancer risks the low value for the DDREF of 1.5 leads to an uncertainty distribution that is skewed towards higher DDREF values and lower REID, which opposes the QF uncertainty estimate which is skewed to higher REID values [2].

Because of the lack of human data following exposure to high LET radiation, animal studies continue to be the main source of information for risk estimates. These studies [16]–[24] suggest that HZE particles and neutrons could produce more lethal tumors compared to tumors from low LET radiation or background tumors, which is a qualitative difference not accounted for in current risk estimates. For low LET radiation there is an implicit assumption made by epidemiology models that the tumors induced by radiation are similar to background tumors in a population. This assumption is consistent with the multiplicative risk model, and also based on lack of information to make an alternative assumption. I considered a sensitivity analysis of the role of increased tumor lethality by adjusting the cancer mortality rates of Eq. (11) for the portion of each particle radiation track that acts in a distinct mode compared to low LET radiation. Also considered is a model (result not shown) where this ratio was adjusted in a tissue specific manner. The lethality fractions in the U.S. population varies substantially with tissue type and such an alternative approach could be further investigated if more information were available. The results of **Table 5** suggests that increased tumor lethality for highly ionizing radiation compared to background or low LET induced tumors that is suggested by animal studies could substantially increase risk estimates and uncertainties estimates for ISS missions, and therefore warrants further study.

QFs are largely based on cellular studies of gene mutation and chromosomal aberrations and limited data on tumor induction in mice from the studies of Fry et al. [16] and Alpen et al. [17] for Harderian gland tumors with a variety of particles. The Harderian gland tumor studies used pituitary implants which acted as tumor promoters such that the effects of radiation exposure was largely on tumor initiation [16], [17]. Therefore information used to estimate radiation QFs are reflective of cancer initiation and provide no information on whether the probability of tumor metastasis is dependent on radiation quality. Alpen et al. [17] showed the tumor response of Fe particles was similar with or without the use of a promoter, which was distinct from the response found for gamma rays.

HZE particle tumor studies reported to date are dissimilar with limitations in particle types, dose and dose-rates, and animal models considered; however are consistent in supporting the hypothesis that HZE particles produce solid tumors in a qualitatively distinct manner compared to low LET radiation, and suggest that RBEs alone do not properly account for potential risks. For leukemia induction, HZE particles appear to be more similar to gamma rays with a low RBE reported [19]. Detailed studies of solid cancers for a variety of particle types and energies and tumor types, with time serial analysis of tumor occurrence and molecular analysis of tumor quality including metastatic potential are needed. These studies should include M and F mice of various strains, including genetically engineered mice, in order to follow-up on the information in studies reported to date [16]–[22].

### Probability of Causation and Early Detection of Cancers

In the past uncertainty analyses of PC estimates have been developed by the National Institutes of Health [28], [29] and in the nuclear industry [30] for terrestrial radiation exposures to screen exposed persons for a potential causal relationship to an observed cancer, and in the determination if monetary compensation should be awarded. The PC predictions reported here suggest that a large portion of cancers that would be observed from astronauts that participated in multiple ISS missions could be attributed to GCR exposure. In contrast, PC predictions for single ISS missions of 6-months are not estimated to be significant. The majority of astronauts would be classified as “healthy workers” based on established evidence of optimal nutrition, exercise, medical care, and abstinence from use of tobacco products. It is well known that NS have lower rates of cancer, circulatory and pulmonary diseases, and longer life-span than former or current smokers [45], [46]. Because cancer risk estimates are made using a mixture of multiplicative and additive risk transfer models, the lower background cancer rates of a NS population reduce radiation risk estimates compared to estimates for the U.S. average population [2], [32]. This leads to the paradoxical result that radiation cancer risks are estimated to be significantly reduced for NS and healthy workers such as astronauts compared to the average U.S. population, while PC estimates for several cancer types are increased. Furthermore, the use of a NS population to represent astronauts may lead to an under-estimation of PC’s, which is suggested by Kaplan-Meir survival analysis and standard mortality ratio (SMR) results reported previously [2]. Results suggest that adjustment for smoking effects relative to the U.S. population does not account for the entire increase in longevity found for astronauts at this time.

Family history and an individual’s possible exposure to other carcinogens should be considered in an assessment of possible causality. The use of family history data should consider the possibility that genetic pre-disposition of specific cancer types [41] may also confer increased radiation sensitivity. Other factors to be considered include smoking history which effects lung, esophagus, oral cavity, bladder and several other cancers, and reproductive history which can impact the risk of breast and other cancers in women [29]. The collection of biodosimetry and biomarker data prior to flight and at multiple time points over several years post-flight [47] would aid in understanding of causation, especially for repeat ISS flyers.

Considerations on the potential role of early cancer detection as a mitigation measure to reduce REID from space radiation exposure lends further support for research studies of HZE particle tumor induction. Radiation has been shown to increase a large number of cancer types (>15) [33]–[35] possibly limiting the benefits from improvements in early detection, which are only available for a few cancer types. For example if ten tissues each contributed 10% to the cancer REID each of which had a background mortality to incidence ratio of 0.5, an improvement of 50% in cure rate through early detection in four cancer types would lead to a reduction in REID of only 20%. However, this result would only be true if space radiation induced tumors were similar to cancers in the general population. Information on HZE particle carcinogenesis is extremely sparse, however animal studies suggest important differences occur. Therefore determining whether or not the etiology of HZE particle and neutron induced tumors are distinct from cancers in the general population or induced by low LET radiation should be a major focus of space radiobiology research. This research not only supports improved risk assessments, but has large implications that could impact possible treatment approaches, studies of individual sensitivity, and considerations of mitigation approaches.

### NCRP and NRC Reviews of Space Radiation Risks and Limits

NASA has been concerned for many years the potential health risks and limitations that could arise from long-term exposure to GCR and possible exposures to sporadic solar particle events. In developing approaches to acceptable risk levels and approaches to quantify and mitigate radiation risks, NASA has sought and received external guidance from the NCRP, and several bodies at the NRC, including the Institute of Medicine (IOM) [48] and Space Science Board (SSB) [49]. Key recommendations with respect to NASA’s regulatory framework for radiation limits, levels of acceptable risk, and approaches to manage uncertainties in risks estimates.

In 1989 the NCRP [4] discussed three types of comparisons of space radiation risks to other risks to form the basis for an acceptable lifetime risk level for space radiation exposure: 1) to limits for ground-based radiation workers, 2) to accidental deaths in the “safe”, “less-safe”, and “hazardous” occupations, and 3) to other accidental fatality risks faced by crew-members. Ground-based radiation workers have lifetime radiation risks no greater than risks experienced on average in the “safe industries”, which was estimated as 0.5% in 1989 [4]. However, the dose limits for ground-based radiation workers were similar to the accidental deaths probability in the “less-safe industries” were life-time risks of up to 3% were estimated at that time. The NCRP noted that because astronauts face other occupational fatality risks, comparison of radiation limits to life-time fatality risks in “hazardous industries” was not appropriate. Based on these observations, the NCRP recommended NASA use a 3% lifetime fatality risk as the basis for dose limits, and for the use of epidemiology based models to estimate age at exposure and sex specific dose limits for astronauts working in LEO [4]. NASA implemented the NCRP recommended sex and age-at-exposure specific dose limits in 1990.

Within a decade of the publication of NCRP Report No. 98, the NCRP reported that the comparison of space radiation risks to the “less-safe industries” no longer supports a 3% fatality risk as a basis for radiation dose limits, because of the improvements in safety leading to lower fatality rates in ground-based occupations [5], [6]. The comparison to less-safe industries would lead to a lower acceptable risk level and stated [5]: *“The NCRP now considers the comparison with lifetime risk associated with the occupational exposure limits recommended for workers on the ground to be the most direct and the most valid. Consequently, the NCRP recommends that the excess lifetime fatal cancer risk due to the radiation exposure of space workers for missions in LEO be limited to three percent excess mortality and that this be the basis for career limits.”* Indeed improvements in ground-based occupational safety since 1989 have occurred with average lifetime fatality risks in most less-safe industries now below 1% [7]. In addition the risk for loss of crew from spaceflight is recently estimated by NASA as 1 in 270 with the NASA Aerospace Safety Advisory Panel (ASAP) suggesting technology investments to reduce this value to less than 1 in 750 [8]. In order to make a comparative risk basis of the 1 in 33 radiation limit to the lower risks for other safety areas, estimates of expected life-loss from radiation risks are needed. In this report we provide estimates of expected life-loss for radiation induced cancer and circulatory diseases following participation in multiple ISS missions.

The IOM noted in their 2001 Safe Passage report [48] and re-affirming an earlier SSB report [49] **, “**Until the radiation hazards to astronauts can be controlled or otherwise mitigated by physical shielding, a 1998 National Research Council report states, *‘long-duration space travel should be postponed (SSB and NRC, 1998)’. Even if an effective physical radiation shield is developed, it in no way diminishes the need for clinical study, including monitoring of crewmembers’ exposures, long-term medical follow-up, and the development of preventive medical treatments to make astronauts more resistant to deep space-induced radiation damage*”. In reviewing the NASA PELs [1], which stated the career radiation limit of 3% Risk of Exposure Induced Death (%REID) at the 95% confidence level (CL), the NRC noted [10], *“The committee strongly recommends that the permissible exposure limits specified in the current NASA radiation protection standards not be violated to meet engineering resources available at a particular level of funding.”* These external safety recommendations to NASA [10], [48], [49] were focused on deep space missions, however it is shown in this report that similar concerns hold for multiple or long-term (1 year or longer) ISS missions especially for missions near solar minimum were dose-rates dominated by GCR can approach 1 mSv/day and about 2-fold higher than missions near solar maximum for similar ISS altitudes.

## Conclusions

The NSCR-2012 models predicts the upper 95% CL for space radiation risk for ISS missions near solar minimum could exceed the NASA REID limit for cancer fatality by 18 months or 24 months for females and males, respectively. Also, median PC and upper 95%-confidence intervals of PC values are predicted to exceed 50% for several cancers for participation in two or more ISS missions of 18-months or longer total duration near solar minimum, or for longer missions at other phases of the solar cycle. Radiation risk estimates will likely continue to be modified as new findings are reported from research studies, especially for highly ionizing radiation, and it is vital that uncertainty analysis be performed to anticipate how such information could modify safety considerations for space missions occurring in the future. The present report suggests that possible qualitative differences related to radiation quality and tumor lethality, and additional risks for circulatory disease from chronic low dose radiation are major issues for astronauts participating in multiple or 1-year ISS missions, as well as for exploration missions to Mars and other deep space destinations.
